# General and Regional Anaesthesia in Cancer Surgery: A 20-year Bibliometric Analysis

**DOI:** 10.4274/TJAR.2025.2083

**Published:** 2026-04-15

**Authors:** Gökçen Kültüroğlu

**Affiliations:** 1 University of Health Sciences Türkiye Ankara Etlik City Hospital, Clinic of Anaesthesiology and Reanimation, Ankara, Türkiye

**Keywords:** Anaesthesia, bibliometric analysis, cancer, metastasis, recurrence

## Abstract

**Objective:**

In recent years, there has been growing interest in the potential effects of anaesthetic agents in cancer surgery. Although the impact of anaesthetic management on long-term oncological outcomes has yet to be definitively established, emerging studies are increasingly exploring interactions with the tumour microenvironment and epigenetic mechanisms. This bibliometric analysis aims to evaluate the existing literature on the use of general and regional anaesthesia in cancer surgery, thereby identifying prevailing trends and informing future research directions.

**Methods:**

This was a retrospective bibliometric study designed to examine publications addressing both “cancer” and “anaesthesia” between 2005 and 2024. A search of the Web of Science database using specified keywords retrieved relevant articles, which were subsequently analysed based on parameters such as publication year, authors, journal, citation count, and country. Data were visualized using software, with network analyses conducted to reveal trends, collaboration networks, and research foci in the literature.

**Results:**

The analysis reviewed 391 articles; the highest number of publications was recorded in 2021 and 2022. These articles collectively garnered 9,068 citations. The most frequently cited studies came from Ireland and the United States, with Dr. Donal Buggy emerging as the leading researcher in the field. The mapping analysis indicated that journals such as Anesthesiology and the British Journal of Anaesthesia were the dominant publication venues.

**Conclusion:**

This study provides valuable insights into the evolving relationship between cancer and anaesthesia over the past two decades. The findings provide a significant foundation for future research and guide scientific development in this field.

**Main Points**

• The potential impact of anaesthetic agents on oncological outcomes in cancer surgery is being increasingly investigated.

• Bibliometric analysis has mapped the main publication trends, key researchers, and collaboration networks in this field.

• Although the United States has produced the highest number of publications on cancer and anaesthesia in the past 20 years, the most prolific and influential author in the field is MD, Donal J. Buggy from Ireland.

## Introduction

Cancer remains a major public health concern worldwide, with approximately 20 million new cases diagnosed each year.^1^ Approximately 65% of cancer patients require at least one surgical procedure for diagnostic or therapeutic purposes during the course of their disease.^2^ In recent years, it has been increasingly suggested that the success of surgical procedures is influenced not only by the surgical techniques used but also by the anaesthetic methods employed. In particular, a growing number of studies have investigated the potential effects of anaesthetic agents on metabolic, neuroendocrine, inflammatory, and immunological responses, highlighting the importance of optimal anaesthetic management in cancer surgery.^3, 4^

It has been hypothesised that anaesthetic agents may, in certain circumstances, interact with tumour biology through mechanisms that either promote tumour progression or, conversely, suppress tumour development.^2^ Volatile anaesthetics have been proposed to suppress the immune response by reducing natural killer (NK) cell activity and increasing levels of pro-inflammatory cytokines [tumor necrosis factor-alpha (TNF-α), interleukin-6 (IL-6), and IL-12], thereby promoting angiogenesis, facilitating tumour cell migration, and potentially supporting tumour progression via these mechanisms. Similarly, recent studies have explored the mechanisms by which opioids may promote tumour growth and have found that they occur either by directly stimulating cancer cells or by enhancing angiogenesis.^5^ In contrast, it has been hypothesised that propofol may exert antiangiogenic effects and preserve immune cell function. Similarly, regional anaesthesia may reduce catecholamine levels through sympathetic blockade and suppress cortisol release by inhibiting the hypothalamic-pituitary-adrenal axis, thereby potentially preventing immunosuppression. Preclinical and some retrospective studies have reported that lidocaine may exert antitumour effects by inhibiting oncogene expression, while non-steroidal anti-inflammatory drugs may limit both inflammation and tumour progression by reducing the expression of inflammatory cytokines (nuclear factor kappa-light-chain-enhancer of activated B cells, TNF-α and IL-6) and suppressing related signalling pathways.^6, 7, 8^ These findings suggest that the choice of anaesthetic technique may influence long-term outcomes after cancer surgery. However, the results of several large-scale clinical studies have failed to support these hypotheses.^9, 10, 11, 12^ Studies to date have consistently suggested that certain routine anaesthetic and analgesic techniques used during cancer surgery have not shown long-term oncological effects.^2^ However, recent studies have focused more on the effects of anaesthesia on interactions with the tumour microenvironment [e.g.,neutrophil extracellular traps (NETs)] rather than on isolating cellular processes.^13^ In addition to NET formation (NETosis), emerging studies indicate that cancer epigenetics may also play a significant role in the cancer-anaesthesia relationship.^14, 15, 16^ Given that large patient cohorts, long-term follow-up of oncological outcomes (e.g., metastasis, disease-free survival, and mortality), and a robust registry system are required to provide definitive evidence and ensure sufficient statistical power, these studies may alter our understanding of the relationship between cancer and anaesthesia.

Bibliometric analysis is essential for assessing the current state of a research field and pinpointing future trends, especially in disciplines that rely on thorough literature reviews. It plays a key role in uncovering critical clusters, landmark studies, and leading centres of expertise within a given domain. As such, it serves as a foundational step enabling researchers to make informed decisions about the areas in which they aim to make advances.^17^ Recent evidence has raised interest in the potential influence of anaesthetic techniques on cancer biology, particularly with regard to tumour recurrence and metastasis. Within this context, the role of general (inhalation or intravenous) versus regional anaesthesia remains a subject of ongoing debate. To systematically explore this area, a bibliometric analysis was conducted on studies specifically addressing these anaesthetic approaches in the context of cancer surgery. The most influential publications, authors, citation networks, and research trends in the field of "anaesthetic techniques and cancer" were evaluated based on the available data, thereby providing researchers in this area with a guiding perspective.

## Methods

### Ethical Statement

The present study was a bibliometric analysis and did not require ethics committee approval because it was conducted using publicly available data.

### Study Design

The present study was a retrospective observational analysis designed to examine the bibliometric characteristics of publications on cancer and general-regional anaesthesia. The Strengthening the Reporting of Observational Studies in Epidemiology guidelines were used for reporting.

### Data Sources and Search Strategy

A search on the study topic of cancer and general-regional anaesthesia was conducted on December 28, 2024, in the Web of Science (WoS) Science Citation Index Expanded (SCIE) database (Clarivate Analytics, Philadelphia, PA, USA; https://www.webofscience.com/wos/). The search was performed by entering the keywords “cancer” and “general” and “regional” and “anaesthesia” into the topic field. The results were limited to publications from 2005 to 2024. Retracted and duplicate articles were excluded. Data were collected using bibliometric parameters, such as title, authors, year of publication, journal, number of citations, and country of origin. The database search strategically selected the keywords ("cancer" and "general" and "regional" and "anaesthesia") to identify studies on anaesthetic techniques most frequently examined in relation to cancer. Although various anaesthesia-related factors may affect cancer outcomes, the analysis focused solely on general and regional anaesthesia.

### Data Extraction and Visualisation

The articles retrieved from the WoS database were exported using the Export Records to Tab Delimited File and Excel formats. The exported data included complete record information related to citations, as well as detailed reference information for each source. Microsoft Excel 2019 (Microsoft Corporation, Santa Rosa, CA, USA) and Visualization of Similarities viewer (VOSviewer) (version 1.6.18; Leiden University, the Netherlands) were used for the analyses. VOSviewer enables the visual representation of bibliographic data and performs network-based analyses. In the figures, the size of the data points represents the number of associated documents, while the colour gradient indicates cluster identity in the network visualisations. The thickness of the lines connecting the data points reflects the strength of the links between them and depends on the analysed subject (e.g., journal, author, or keyword).

### Statistical Analysis

No inferential statistical analysis was performed in this study. Descriptive bibliometric indicators — including total citations, average citations per article, and the h-index — were calculated from the bibliographic data obtained from WoS. Network analyses (co-authorship, co-citation, and keyword co-occurrence) were conducted using VOSviewer (version 1.6.18) and tabulated using Microsoft Excel 2019.

## Results

A total of 419 articles were identified by searching the WoS database using the keywords. After restricting the time frame to 2005-2024, 393 articles remained. Following the exclusion of retracted (n=1) and duplicate (n=1) articles, 391 articles were included for further analysis.

### Number of Publications and Trends

**Figure 1:**
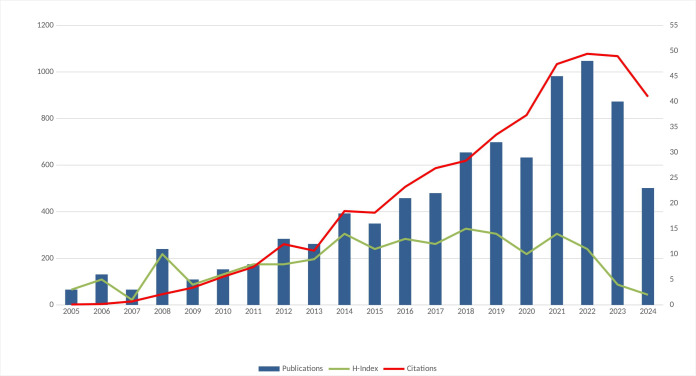
Annual distribution of publications and citations in the literature.

The number of articles on the study topic increased after periods of stagnation that typically lasted approximately three years between 2005 and 2024. Evaluation of publication numbers showed that 2022 marked the peak, with 48 publications and 1,078 citations (Figure 1). An analysis of fluctuations and periods of stability over the past two decades revealed that the overall trend in publication volume remained relatively steady. Based on the growth regression model represented by the equation y=3.298·e^(0.097·t)^, approximately 23 publications in this field are projected for 2025. Overall, these findings suggest a decline in research activity on regional and general anaesthesia in cancer surgery.

According to the citation analysis, the total number of citations for the 391 publications was 9,068. After self-citations by the authors were excluded, 7,885 citations were identified. The average number of citations per publication was 23.13, and the H-index was 47. Of the publications, 96% were written in English, and 75% were indexed in the SCIE database (Table 1).

The first article published in 2005, marking the beginning of the search strategy, appeared in Anesthesia & Analgesia (Journal Citation Indicator: 2.04, Q1) under the title “Inhibition of the stress response to breast cancer surgery by regional anesthesia and analgesia does not affect vascular endothelial growth factor and prostaglandin E2”*.* This study, authored by O’Riain SC, Buggy DJ, Kerin MJ, and colleagues, has received 96 citations. The corresponding author is MD, Seosamh O’Riain, and the study was conducted in the Department of Anesthesia at Mater Misericordiae University Hospital, Dublin, Ireland.^18^ The details of the top five most-cited articles according to the search criteria are summarised in Table 2. The most cited article (n=650) was published in Anesthesiology (Journal Citation Indicator: 3.43, Q1) in 2006. The study, titled “Can anesthetic technique for primary breast cancer surgery affect recurrence or metastasis?” was authored by Exadaktylos AK, et al.^19^. The corresponding author was MD, Donald J. Buggy, and the study was conducted at the Department of Anesthesia, Mater Misericordiae University Hospital, Dublin, Ireland.

Analysis of the clinical studies included in the research data revealed that breast cancer was the most frequently investigated malignancy, representing 63.3% of the studies. This was followed by lung cancer, which accounted for 14.6%, and prostate cancer at 10.2%, highlighting the relative focus of current clinical research on these cancer types.

**Figure 2:**
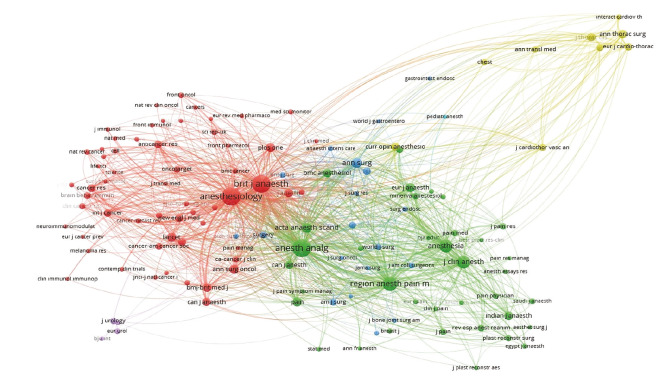
VOSviewer co-citation network visualisation. VOSviewer, visualization of similarities viewer.

Based on the title data of publications in the WoS database, an analysis of journals containing sources cited at least 20 times identified six clusters using the VOSviewer mapping technique. The strongest cluster (Cluster 1) included 59 journals, the most prominent of which were Anesthesiology and the British Journal of Anesthesiology. Cluster 2 features notable journals such as Anesthesia & Analgesia, Regional Anesthesia and Pain Medicine, Journal of Clinical Anesthesia, and Anaesthesia (Figure 2).

### Keyword Analysis

**Figure 3:**
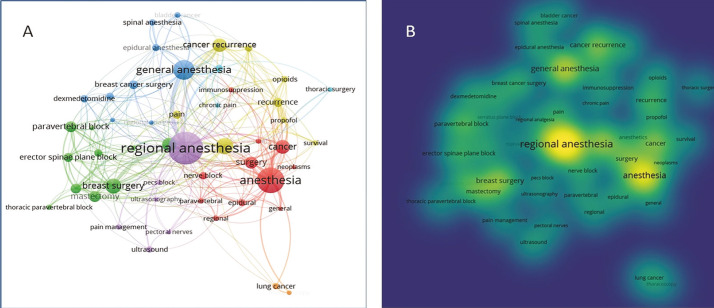
A) VOSviewer keyword network visualisation, B) VOSviewer keyword density visualisation (the depth of colour is proportional to the frequency of occurrence). VOSviewer, visualization of similarities viewer.

A visualisation analysis of all included publications was performed using 49 keywords that appeared more than five times, resulting in seven clusters (Figure 3). Keywords such as “anaesthesia”, “cancer”, “regional anaesthesia”, and “general anaesthesia” were frequently used because they represented the main topics of the study. Other notable keywords were “breast surgery”, “breast cancer surgery”, “mastectomy”, and “cancer recurrence”.

### Country Analysis

According to an analysis of WoS articles over the past 20 years, the five countries with the highest number of publications in the field of cancer and anaesthesia were the US (n = 101), China (n = 53), Italy (n = 36), India (n = 33), and Germany (n = 29). However, the countries with the highest number of citations were the US (n = 4,998), Ireland (n = 2,169), and China (n = 934). The US ranks highest in terms of both publication volume and citations. Notably, while Ireland lags behind other countries in terms of publication output, it ranks second (after the US) in citation count.

### Institution and Author Analysis

The five most productive authors on the research topic are listed in Table 3. The author with the highest number of publications and citations is Dr Donald J. Buggy. Citations for articles authored by those listed in Table 3 accounted for 37.21% of all citations in the research area.

## Discussion

In the present study, a bibliometric analysis was conducted of articles published over the last 20 years that combined the terms “general and regional anaesthesia” and “cancer”. The findings revealed that the years 2021 and 2022 had the highest number of articles published, with English the dominant language of the articles. The US was the leading country in terms of both publication volume and citations. The author with the most publications and citations in the research field was Prof. MD, Donald J. Buggy, based in Ireland. The most frequently cited articles were published in Anesthesiology and the British Journal of Anesthesiology.

Although the impact of anaesthetic and analgesic methods on cancer outcomes was first raised 40 years ago,^23^ it was not until 2006 that an observational study highlighted this issue.^19^ In the highly cited study published by Exadaktylos et al.,^19^, the authors investigated whether the choice of anaesthetic technique in breast cancer surgery influences the risk of cancer recurrence or metastasis. Their findings suggest that patients who received paravertebral anaesthesia and analgesia had a significantly lower risk of recurrence or metastasis compared with those managed with general anaesthesia and morphine-based analgesia alone. The authors hypothesised that preservation of immune function, achieved through attenuation of the surgical stress response and reduced opioid consumption, may underlie this potential benefit. This retrospective study also emphasises the need for prospective clinical trials to elucidate the impact of regional anaesthesia on long-term oncological outcomes.^19^ Another highly cited study retrospectively examined the effect of anaesthetic technique on cancer recurrence in patients undergoing radical prostatectomy. The authors reported that the combination of epidural anaesthesia and analgesia with general anaesthesia significantly reduced the risk of recurrence compared with general anaesthesia and postoperative opioid use alone. It was further suggested that regional anaesthesia may help preserve immune function by attenuating the surgical stress response, reducing anaesthetic requirements, and minimising opioid exposure.^20^ In addition, a highly cited review examines the potential effects of surgical and anaesthetic techniques, as well as other perioperative factors, on cancer recurrence. It primarily emphasises the concept that surgery and the anaesthetic methods employed may weaken the patient’s immune system, thereby facilitating the dissemination and metastasis of cancer cells. Particular attention is drawn to the critical role of cellular immune mechanisms, such as NK cell activity, in the postoperative period. The review discusses the potential influence of intravenous and volatile anaesthetics, local anaesthetics, opioids, and non-steroidal anti-inflammatory drugs, as well as other factors including blood transfusion, pain, stress, and hypothermia, on cancer prognosis. It also suggests that evidence supporting the potential benefits of regional anaesthesia is steadily increasing.^21^

These and many similar studies have focused on the effects of anaesthetics on the behaviour of cancer cells. While preclinical research in this area is highly compelling, clinical findings remain inconclusive and continue to be a subject of debate.^24, 25, 26, 27, 28, 29^ This may be because cancer is not a single disease, and cancer types and subtypes may respond differently to therapeutic interventions. Another reason is that patient-specific tumour genomic alterations may be the primary determinants of the relationship between a drug and its outcomes.^30^ Genomic research suggests that, beyond cancer subtypes, interpatient differences in tumour genomes could modify the dose-response relationship between intraoperative opioid dosage and tumour recurrence. In patients with specific tumour types, such as those with lung or colon adenocarcinoma, a relationship between opioid dosage and recurrence has been identified.^14, 31^ A transcriptomic study of renal cell carcinoma from The Cancer Genome Atlas Program, a large cancer-genomic research initiative, has identified gene networks, associated with survival, that can be modulated by opioids.^32^ Differences in the expression of these tumour gene networks have also been shown to affect individual patient outcomes in response to intraoperative opioid doses.^33^ Importantly, this finding is not limited to opioids, but also applies to all anaesthetic and analgesic agents.^34^ From this perspective, supporting our anaesthesia and analgesia interventions with tumour-specific genomic sequencing and data on their effects on cancer subtypes seems to pave the way for personalised treatment. This suggests that additional research is likely to be conducted on the relationship between cancer and anaesthesia. At this point, recognising the leading papers, authors, institutions, and countries related to this topic will help guide new researchers and other readers interested in this field.

Although numerous publications included in this bibliometric analysis have reported varying results over the years, current evidence presents a consistent picture regarding the impact of anaesthetic techniques on long-term oncological outcomes. A comprehensive review published in Anesthesia & Analgesia in April 2025, with contributions from Donal Buggy and other leading researchers in the field, summarised nearly two decades of research and demonstrated that, apart from peritumoural lidocaine infiltration, most anaesthetic approaches exert a neutral effect on cancer recurrence and metastasis.^2^ Similarly, a recent review reported that regional anaesthesia has a neutral effect on oncological outcomes following tumour resection, and evidence regarding propofol-based total intravenous anaesthesia versus volatile anaesthesia is also tending towards a neutral effect.^35,36^ While preclinical studies continue to provide mechanistic insights, the precise pathways through which anaesthetics may influence oncological processes, including patient-specific tumour genomics and tumour subtypes, remain unclear. Future research may further elucidate the role of genetic variability and epigenetic mechanisms in the interaction between anaesthetics and cancer biology.

### Study Limitations

The present bibliometric study provided information on the prevalence of articles related to ‘general and regional anaesthesia with cancer’, the journals in which the most frequently cited papers are published, the authors who have contributed to this field and the institutions they are affiliated with. However, this study has some limitations. First, as it relied solely on the WoS database, relevant publications indexed in other databases may have been overlooked. Second, recently published studies might not have been extensively cited due to their shorter publication timelines. Third, one limitation of our study is the restricted set of search terms employed (‘cancer’ and ‘general’ and ‘regional’ and ‘anaesthesia’). While this strategy allowed us to maintain a clear focus on anaesthetic techniques in cancer surgery, it may have led to the exclusion of potentially relevant articles that did not explicitly include these keywords. Therefore, interpretation of the findings should be considered within the context of this methodological constraint. Finally, the study provided only the quantitative characteristics of the publications on the research topic. Despite these limitations, the present study provided valuable reference material for understanding global research trends and focal points regarding general and regional anaesthesia and cancer.

## Conclusion

In the present study, biomedical research trends in general and regional anaesthesia in cancer over the past 20 years were analysed using bibliometric methods. The most influential papers, researchers, journals, institutions and countries that had shaped the scientific development of the field were identified, and their contributions were examined in detail. The findings provide an important reference to guide future research in the field and to inform strategic planning of scientific studies.

## Ethics

**Ethics Committee Approval:** The present study was a bibliometric analysis and did not require ethics committee approval because it was conducted using publicly available data.

**Informed Consent:** Due to the retrospective nature of the study, an informed consent was not required.

## Figures and Tables

**Table 1. Language, Index Coverage, and Citation Information of Articles:** 

**Language**	**n**	**Index**	**n**	**Citation**	**n**
English	375	SCIE	295	Citing article	5,227
German	6	ESCI	88	Citing article (excluding self-citations)	4,969
Spanish	6	Conference	6	Number of citations	9,068
French	3	Proceeding	1	Number of citations (excluding self-citations)	7,885
Portuguese	1	Book chapter	1	H-index	47

SCIE, Science Citation index Expanded; ESCI, Emerging Sources Citation index; H-index, Hirsch index

**Table 2. Most Cited Articles Based on Search Criteria:** 

-	**Publication**	**First three authors**	**Published journal**	**Year**	**Number of citations**
1	Can anesthetic technique for primary breast cancer surgery affect recurrence or metastasis?^19^	Exadaktylos AK. Buggy DJ. Moriarty DC.	Anaesthesiology	2006	650
2	Anesthetic technique for radical prostatectomy surgery affects cancer recurrence - a retrospective analysis.^20^	Biki B. Mascha E. Moriarty DC.	Anaesthesiology	2008	478
3	Effect of anaesthetic technique and other perioperative factors on cancer recurrence.^21^	Snyder GL. Greenberg S.	British Journal of Anesthesiology	2010	373
4	The effect of anesthetic technique on survival in human cancers: a meta-analysis of retrospective and prospective studies.^22^	Chen WK. Miao CH.	PLoS One	2013	171
5	Can anaesthetic and analgesic techniques affect cancer recurrence or metastasis?^5^	Heaney A. Buggy DJ.	British Journal of Anesthesiology	2012	166

**Table 3. Authors with the Most Publications and Citations, and Their Affiliated Institutions:** 

**Author**	**Number of publications**	**Number of citations**	**Institution**	**Country**
Donal Buggy	16	1229	Mater Misericordiae University Hospital	Ireland
Daniel I. Sessler	8	916	Cleveland Clinic	US
Juan Cata	6	113	MD Anderson Cancer Center	US
Ming-Hui Hung	5	94	National Taiwan University Hospital	Taiwan
Alan D. Kaye	5	60	Louisiana State University	US
